# The cost of amino acid catabolism for energy utilization in broiler chickens

**DOI:** 10.1016/j.psj.2025.105199

**Published:** 2025-04-19

**Authors:** Shemil P Macelline, Peter H Selle, Mehdi Toghyani, Sonia Y Liu

**Affiliations:** a School of Life and Environmental Sciences, Faculty of Science, The University of Sydney, Camperdown, NSW 2006, Australia; b Poultry Research Foundation, The University of Sydney, Camden, NSW 2570, Australia; c Sydney School of Veterinary Science, The University of Sydney, Camperdown, NSW 2006, Australia

**Keywords:** Amino acid catabolism, Broiler, Energy, Lipogenesis

## Abstract

This review highlights that utilization of dietary amino acids for energy metabolism in broiler chickens imposes a metabolic cost, as their primary role is to support body protein synthesis. This issue becomes more critical in reduced-crude protein (CP) diets. When amino acids are used as fuel for enterocytes or undergo catabolism in the liver, they are diverted from body protein accretion. Catabolism of amino acids for energy generates α-keto acids and ammonia. α-Keto acids can be fully oxidized to produce ATP or converted into pyruvate, ketone bodies, and intermediates of the tricarboxylic acid cycle. Meanwhile, ammonia must be detoxified through the uric acid cycle, a process that requires energy, glycine, and aspartic acid. Derivatives of α-keto acids can contribute to gluconeogenesis and *de novo* lipogenesis, leading to glucose and fatty acid synthesis, respectively. The α-keto acid derivatives are more likely to undergo *de novo* lipogenesis in broilers, as evidenced by consolidated data in this review. However, *de novo* lipogenesis is also an energy-intensive process. Therefore, enhancing the efficiency of dietary amino acid conversion to body protein requires reducing their utilization for energy metabolism. This may be achieved through dietary manipulations, as previous studies indicate that amino acid catabolism in enterocytes and the liver is influenced by starch and protein digestive dynamics, dietary amino acid compositions, and the primary feed grain used in diets. In reduced-CP broiler diets, supplementation of glutamic acid and potentially glutamine, aspartic acid, and proline could mitigate the catabolism of essential amino acids in enterocytes. Additionally, moderating starch digestion rates may reduce amino acid catabolism in enterocytes. Moreover, optimizing the balance of dietary protein-bound and non-bound amino acids could minimize amino acid catabolism in the liver. In summary, reducing the contribution of amino acids to energy metabolism in broiler chickens is particularly beneficial in reduced-CP diets, ultimately supporting more sustainable chicken meat production.

## Introduction

The role of dietary protein in energy metabolism, rather than its conversion into body protein, should be carefully considered to optimize production traits in livestock animals (Miller, 2004). In broiler chickens, the energy derived from dietary protein has been discussed in the context of *de novo* lipogenesis (Mizuno et al., 1982) and its use as a fuel for enterocytes (He et al., 2018; He and Wu, 2022). However, protein-based energy metabolism is widely regarded as an inefficient and costly process in livestock animals (Blaxter, 1989; Miller, 2004; Musharaf and Latshaw, 2019).

The gross energy of proteins in different feedstuffs varies based on their chemical composition, particularly the proportion of carbon, hydrogen, and oxygen, as well as their amino acid sequences (Merrill and Watt, 1955). Similarly, the heat of combustion of protein in cereals, legumes, and meat has been reported to be 5.80, 5.70, and 5.65 kcal/g, respectively (Atwater and Bryant, 1900). Moreover, the energy content of individual amino acids varies significantly, ranging from 1,362 to 6,740 kcal/kg, as reported in the literature (Tillman, 2019; Boisen and Verstegen, 2000; Pesti et al. 2024). For amino acids to contribute to energy metabolism, they must first undergo catabolism, a process that separates the amine group from the α-keto acid. The metabolic fate of the α-keto acids depends on the carbon skeleton of specific amino acid. These α-keto acids can enter glucogenic pathways, ketogenic pathways, or both, or they can be completely oxidized to produce ATP in animal tissues (Hayamizu, 2017). The liver is the primary site of amino acid catabolism, where α-keto acids are metabolized through glucogenic or ketogenic pathways. In the glucogenic pathway, they are converted into pyruvate or intermediates of the tricarboxylic acid cycle, which can also be used for glucose synthesis. In the ketogenic pathway, they are converted into ketone bodies, which may subsequently be metabolized into fatty acids. In poultry, amino acid catabolism also generates ammonia, which is excreted as uric acid. This process incurs an energy cost, as approximately 34.4 kJ is lost for every gram of nitrogen excreted as uric acid, contributing to the gross energy of the excreta (Hill and Anderson, 1958, McNab, 2000). Excessive use of amino acids for energy results in increased heat production and nitrogen excretion, representing an unnecessary metabolic and economic cost to broiler production.

The digestive dynamics of protein and starch may be defined as a three-phase progression comprising digestion, absorption and transition of protein/amino acids and starch/glucose from the gut lumen to the portal circulation (Liu and Selle, 2017). Even though intestinal tissue represents only about 5 % of the body weight, gastrointestinal tract alone is estimated to consume approximately 20 % of dietary energy in animals, with the energy demand in enterocytes primarily accounted on protein turnover and ion exchange (Cant et al., 1996, McBride and Kelly, 1990). In poultry, gastrointestinal tract accounts for approximately 10.3 % of total energy expenditure while gut tissue represents about 1.69 % relative weight to body weight (Spratt et al., 1990). Moreover, chickens exhibit higher oxygen consumption by their gastrointestinal tract compared to mice, rats, sheep, and cattle, despite broiler intestine representing a relatively small proportion of their body weight than other animals (Cant et al., 1996). The demand for amino acids as an energy substrate in enterocytes has been discussed in pigs (Stoll et al., 1998) and poultry (Wu et al., 2005; He et al., 2018). A significant portion of dietary amino acids absorbed in the small intestine is unlikely to reach the portal circulation, as they are extensively utilized within the gut mucosa through anabolic and catabolic pathways (Stoll et al., 1998). The extent of dietary amino acid catabolism in enterocytes is likely to depend primarily on starch digestion rates (Van der Meulen et al., 1997; Yin et al., 2019) and the source of the amino acids (protein-bound *versus* non-bound) (Eugenio et al., 2023).

Understanding the metabolic fates of amino acids in liver and enterocytes is pivotal to optimising digestive dynamics and net protein deposition in broiler chickens. Therefore, this review discusses the consequences of using dietary amino acids as an energy substrate in broiler chickens and explores possible dietary strategies to minimise amino acid catabolism, thereby enhancing dietary protein utilization for overall growth performance and productivity.

## Dietary protein conversion

The efficiency of converting dietary protein into tissue protein in 42-day-old broiler chickens was reported as 41 % by Baker (2009), while Wilkinson (2011) calculated the efficiency of converting dietary protein into edible meat over 42-day post-hatch period as 33 %. A retrospective analysis of three studies conducted at the Poultry Research Foundation estimated the proportion of dietary amino acids deposited in the *Pectoralis* muscle at 35 days post-hatch, as summarized in Table 1. On average, the efficiency of dietary amino acid conversion into *Pectoralis* muscle was estimated as 18.8 %. This indicates a considerable amount of dietary amino acids do not incorporate into body protein.

**Table 1:** Estimated proportions (%) of dietary amino acids deposited into *Pectoralis* muscle at 35 days post-hatch.

Item	Macelline et al. (2023a)		Macelline et al. (2023b)		Macelline et al. (2023c)				Average ± SD
Dietary-CP, g/kg	210^1^	180^1^	210^1^	170^1^	210^1^	170^1^	210^2^	170^2^	
Arginine	15.9	16.3	17.2	16.1	19.6	17.4	18.4	18.4	17.4 ± 1.31
Histidine	24.9	25.6	31.1	34.4	28.8	30.8	26.5	31.1	29.2 ± 3.29
Isoleucine	13.7	13.8	15.8	15.4	16.8	16.0	14.6	15.1	15.2 ± 1.08
Leucine	16.6	15.8	19.2	18.5	19.5	18.6	13.4	15.4	17.1 ± 2.17
Lysine	19.5	18.3	23.0	21.8	23.9	21.0	21.9	22.6	21.5 ± 1.85
Methionine	21.7	21.7	23.0	16.2	25.8	18.6	24.6	19.3	21.4 ± 3.20
Phenylalanine	12.4	13.7	13.1	12.3	14.3	16.4	12.5	14.4	13.6 ± 1.39
Threonine	17.7	17.2	17.6	17.0	19.6	18.0	17.8	18.5	17.9 ± 0.82
Valine	12.8	11.6	14.7	13.8	15.5	13.9	14.1	14.2	13.8 ± 1.18
Alanine	20.9	26.8	26.2	43.0	26.0	40.4	21.6	17.3	27.8 ± 9.20
Aspartic acid	18.1	30.5	21.0	49.3	19.8	45.4	15.9	26.0	28.3 ± 12.69
Glutamic acid	8.87	8.60	9.55	11.8	10.7	11.4	12.2	15.8	11.1 ± 2.32
Glycine	14.0	14.0	17.8	12.2	18.2	12.3	16.8	14.0	14.9 ± 2.37
Proline	6.40	6.10	8.10	9.51	9.16	8.46	10.3	12.2	8.8 ± 2.00
Serine	13.7	14.0	16.3	25.0	17.1	24.3	15.8	22.4	18.6 ± 4.61
Tyrosine	21.7	19.4	25.0	23.9	29.3	11.01	26.6	34.3	23.9 ± 6.94
Average	16.2	17.1	18.7	21.3	19.6	21.3	17.7	19.4	18.8 ± 1.86
Final body weight (g)	2373	2149	2653	2362	2743	2519	2823	2638	2533 ± 225.6
*Pectoralis* weight (g)	488	404	638	523	655	550	635	553	484 ± 187.6
*Pectoralis* yield (g/kg)^3^	206	188	241	221	239	218	225	210	218 ± 17.4
FCR^4^	1.455	1.606	1.411	1.477	1.387	1.511	1.369	1.437	1.457 ± 0.0762

1 Diets were based on wheat.

2 Diets were based on sorghum.

3 *Pectoralis* weight relative to body weight.

4 FCRs were determined for 14 to 35 days post-hatch.

There is lack of data on the proportion of dietary protein used for energy metabolism in broiler chickens. One possible approach to estimate the proportion of amino acids used in energy metabolism is to measure uric acid excretion. During amino acid catabolism, removal of the amine group generates ammonia (NH₃), a toxic compound for broiler chickens (Selle et al., 2024; Stern and Mozdziak, 2019). NH₃ is detoxified by condensing with glutamic acid to form glutamine, which subsequently enters the uric acid cycle in broilers. This process ultimately converts NH₃-N into uric acid-N, which is excreted (Selle et al., 2023; Van Milgen, 2021). Measuring uric acid-N in excreta thus provides an indication on the extent of dietary amino acids diverted for energy metabolism. However, this method has limitations because not all NH₃ in the body is converted to uric acid, and not all α-keto acids is used for energy metabolism.

The proportion of uric acid-N to dietary-N in broiler chickens has been quantified in Selle et al. (2021a), ranging from 6.9 % to 17.7 % across dietary treatments. These findings highlight that amino acid contributions to energy metabolism are broadly influenced by diet compositions. However, utilizing amino acids for energy metabolism is inefficient and more costly compared to carbohydrates or fats (Blaxter, 1989; Musharaf and Latshaw, 2019). This inefficiency arises because energy production from amino acids depletes building blocks for protein synthesis, increases nitrogen excretion, and incurs additional energy costs for uric acid synthesis. For instance, excreting 1 g of nitrogen as uric acid requires 64.7 kJ of metabolizable energy (Van Milgen, 2021). Also, production of 1 mole of uric acid consumes 1 mole of glycine (Salway, 2018). Furthermore, energy production from amino acids results in higher heat production compared to carbohydrates or fats (Musharaf and Latshaw, 2019). Forbes et al. (1944) demonstrated that protein as the sole dietary energy source resulted greater heat increments in rats compared to a diet containing a combination of proteins, carbohydrates and fats. Boisen and Verstegen (2000) have been tabulated the ATP production at cellular level from amino acids, carbohydrates and lipids as physiological energy. Consequently, energy utilisations were calculated by using the ratio of physiological energy to gross energy where amino acids exhibited lower and more variable utilization (48 %) than carbohydrates and fat (67 %). Energy utilization in amino acids varied from 32 % for histidine to 60 % for isoleucine and glutamic acid with 8.81 % standard variation. The plausible explanation for the low energy utilization and variability in amino acids may be attributed to the energy cost associated with urea or uric acid synthesis and the differing number of amine groups present in individual amino acids, respectively. Moreover, in broiler chickens, high-protein diets have been associated with reduced energy efficiency (Westerterp-Plantenga, 2008). This inefficiency could be supported by findings from Selle et al. (2021a), where a diet containing 222 g/kg CP resulted in a higher ratio of uric acid-N to dietary-N than a diet containing 165 g/kg CP by 38 % (15.1 % versus 9.3 %). This higher uric acid-N excretions could be associated with the significant reductions of ME:GE ratios in 222 g/kg-CP diets than 165 g/kg-CP counterparts in maize- or wheat-based diets (Chrystal et al., 2021). Additionally, the consequences of utilizing amino acids as an energy source maybe evident in the linear correlations reported in Selle et al. (2021a). In this study, proportions of uric acid-N relative to dietary-N were negatively correlated with weight gain (r = -0.587; P = 0.010) and positively correlated with feed conversion ratio (FCR) (r = 0.635; P = 0.005).

## Amino acid contributions for energy metabolism in broiler chickens

### Energy metabolism in enterocyte

The general consensus is that both glucose and amino acids are oxidized in the gut mucosa to meet the energy demand of the gastrointestinal tract which is estimated to be approximately 20 % of the ingested energy (Cant et al., 1996). Amino acid catabolism in the gut mucosa is nutritionally regulated, and post-enteral amino acid availability may improve if intestinal energy is primarily derived from glucose (Yin et al., 2019). Dietary amino acid catabolism in enterocytes accounts for approximately 20 %, as indicated by the net portal outflow of NH₃, which represents 18 % of the total amino acid nitrogen in diets in young pigs (Stoll et al., 1998). They concluded that the catabolism of essential amino acids by mucosal cells is quantitatively greater than their incorporation into mucosal protein. Moreover, it has been reported that the majority of dietary glutamine (Gln), glutamate (Glu), and aspartate is catabolized in enterocytes and therefore not available to extraintestinal tissues (Battezzati et al., 1995; Stoll et al., 1998). However, there is uncertainty as to which amino acid is primarily used as the dominant enterocyte fuel. Lacey and Wilmore (1990) reported that glutamine (Gln) appears to be a unique amino acid that serves as a respiratory fuel for enterocytes, while Fleming et al. (1997) concluded that glucose and Gln are the main energy substrates in rat enterocytes. However, contrast findings were reported that glutamate (Glu) is catabolised to a greater extent than Gln in enterocytes in Reeds et al. (2000). Moreover, findings of Burrin and Stoll (2009) stated that Glu is the most catabolizable amino acid in gut mucosa. According to the rate of CO_2_ production in broiler enterocytes, Glu catabolism is 10 times higher than that from glucose while the rate of CO_2_ production from Gln and fatty acids was much lower than Glu (He et al., 2018). Moreover, it has been stated that chicken enterocytes have low phosphate-activated glutaminase activity which limited the conversion of Gln into Glu to complete oxidation (Wu et al., 1995a; Wu et al., 1995b). The extensive catabolism of Glu by the enterocytes should reduce the entry of dietary Glu into the portal circulation in broilers. Likewise, in plasma amino acid concentrations, Glu concentrations are usually lower (<100 µM) compared to Gln (Watford and Wu, 2005). Additionally, in portal (anterior mesenteric vein) circulation of broiler chickens, the mean Gln concentration was found to be 81.7 % higher than Glu (109 versus 60 µg/mL) in Moss et al. (2018). Catabolism pathway of Glu in chicken enterocytes produces alanine, aspartate and α-ketoglutarate where further metabolism of alanine and aspartate are limited but α-ketoglutarate can further be oxidized to CO_2_ in chicken enterocytes via the Krebs cycle to generate energy (He and Wu, 2022).

Despite Glu being the major energy source in broiler enterocytes, other amino acids are also involved in energy provision to enterocytes up to distinct extents. Likewise, it has been stated that branched-chain amino acids are oxidized in enterocytes, with leucine oxidation being 15 and 85 % higher than isoleucine and valine, respectively, in mammalian and avian enterocytes (Furukawa et al., 2021; Wu et al., 2005). The review of Wu (1998) recapped that enterocyte catabolized arginine, proline, branch-chained amino acids, methionine, lysine, phenylalanine, threonine, glycine and serine in diets that accounted 30 – 50 % of dietary amino acids, which are not available for extraintestinal tissues.

The utilization of Glu as the primary energy substrate in enterocytes presents a challenge in reduced-CP diets, as dietary CP reductions significantly lower dietary Glu concentrations. Based on 13 feeding studies, Macelline et al. (2024) estimated that reducing dietary CP from 211 to 172 g/kg decreased dietary Gln + Glu concentrations by 23.4 %. This is primarily because conventional reduced-CP diet formulations do not include non-bound Glu or Gln, and there is a lack of established recommendations for their dietary inclusion in broiler chickens. Consequently, reduced-CP diets with low Glu + Gln concentrations may pose a risk, as essential amino acids could undergo oxidation in enterocytes to meet energy demands.

### *De novo* lipogenesis from amino acids

Typically, broiler chickens on high-starch diets tend to exhibit higher abdominal fat deposition compared to those on low-starch, iso-caloric diets, with same energy intakes, as discussed in Selle et al., (2022a). The underlying mechanism may be *de novo* lipogenesis, triggered by a postprandial surplus of glucose derived from starch. This phenomenon is particularly relevant in avian species, as they have limited muscle glycogen stores (Braun and Sweazea, 2008). Likewise, a transcriptomic analysis exhibited upregulation of *de-novo* lipogenesis related genes in broilers offered low-CP, higher starch diets than those offered conventional diets (Asiamah et al., 2025).

Mizuno et al. (1982) suggested that in broilers fed high-CP, low-starch diets, hepatic fatty acid synthesis may primarily originate from amino acids rather than glucose. The study examined the influence of dietary glucose and an amino acid mixture on hepatic fatty acid synthesis in White Leghorn cockerels and found that, in low-CP, high-starch diets, fatty acid synthesis from glucose was 12.2 times higher than from amino acids (4.65 % vs. 0.38 %). Conversely, in high-CP, low-starch diets, fatty acid synthesis from amino acids was 8.75 times higher than from glucose (0.70 % vs. 0.08 %). Furthermore, the overall conversion of glucose or amino acids to fat was reported to be greater in low-CP diets compared to high-CP diets. This may be because the carbon skeletons derived from amino acid catabolism in broiler chickens are more likely to be directed toward lipogenesis (Geraert et al., 1990). The outcomes of Crown et al. (2015) provision that branched chain amino acid catabolism associated with fatty acid synthesis in adipocytes. Additionally, findings by Saunderson and Whitehead (1987) showed that amino acid catabolism is higher in fat-line broilers than in their lean counterparts, which may indicate the conversion of amino acid carbon to fatty acids via *de novo* lipogenesis. Furthermore, plasma concentrations of glucogenic amino acids have been reported to be 10–15 % lower in broilers selected for fat deposition compared to those selected for lean muscle growth (Geraert et al., 1987). Notably, a retro-analyses of data obtained from three research studies completed at Poultry Research Foundation is shown in Table 2. Consequently, specific plasma concentration of certain glucogenic amino acids exhibited negative correlations with relative abdominal fat-pad weights in broiler chickens. Moreover, plasma concentrations of these amino acids exhibited positive correlations with weight gain, indicating that glucogenic amino acids not utilized for protein synthesis may contribute to *de-novo* lipogenesis in broiler chickens after their catabolism. Whitehead and Saunderson (1987) suggested that lipogenesis pull the amino acids away from protein synthesis.

**Table 2:** Pearson correlation coefficients between individual plasma amino acid concentrations and abdominal fat-pad weights or weight gains in broiler chickens across three independent studies conducted at the Poultry Research Foundation.

Study	Amino acid	Abdominal fat-pad weight		Weight gain	
		r =	P =	r =	P =
1Macelline et al. (2023c)	Histidine	-0.319	0.045	0.317	0.047
	Proline	-0.330	0.038	-	NS
	Cystine	-0.321	0.044	-	NS
Macelline et al. (2023a)	Asparagine	-0.517	0.034	0.678	0.003
	Aspartic acid	-0.530	0.029	0.797	<0.001
	Alanine	-0.496	0.043	0.724	<0.001
	Cystine	-0.580	0.015	0.601	0.011
Macelline et al. (2023b)	Asparagine	-0.563	0.008	0.571	0.007
	Arginine	-0.524	0.012	0.708	<0.001
	Isoleucine	-0.604	0.003	0.659	0.001

Pearson correlation coefficients were calculated using individual bird data from each study, relating plasma amino acid concentrations to corresponding abdominal fat-pad weights and body weight gains. Only amino acids showing statistically significant correlations (P ≤ 0.05) with either parameter is presented, unless otherwise noted as NS.

1 Correlations obtained for arginine were not considered in Macelline et al. (2023c) since dietary arginine is an independent variable in this study. NS = non-significant (P > 0.05).

## Factors influencing on energy metabolism from amino acids in broiler chickens

### Starch digestion rates

The utilization of amino acids as an energy substrate in enterocytes may be influenced by the starch digestion rates of the feed grain used in the diets (Yin et al., 2019). Slowly digestible starch has been reported to reduce amino acid catabolism in enterocytes compared to rapidly digestible starch in pigs (Van der Meulen et al., 1997). In this study, although both starch types had similar digestibility, the net portal glucose flux was higher for rapidly digestible starch than for slowly digestible starch. Interestingly, the net portal flux of amino acids was greater in diets containing slowly digestible starch. This suggests that glucose from slowly digestible starch is more extensively utilized by enterocytes than glucose from rapidly digestible starch. Consequently, reduced amino acid catabolism in enterocytes, as glucose from slowly digestible starch likely serves as a key energy substrate to enterocytes. Moreover, outcomes of Enting et al. (2005) and Weurding et al. (2003) indicative that slowly digestible starch reduced amino acid catabolism in enterocytes of broiler chickens. Since Glu is recognized as the most catabolizable amino acid in enterocytes (He and Wu, 2022), the activity of glutamic-oxaloacetic transaminase (GOT) serves as an indicator of the extent of amino acid catabolism within enterocytes. Moreover, most of the amino acids, particularly glucogenic amino acids, are converted to glutamate during their catabolism (Burrin and Stoll, 2009). Yin et al. (2019) reported a trend (P = 0.057) towards increased GOT activity in the ileum with the transition from slowly digestible dietary starch to rapidly digestible starch. Starch digestion rates in commonly used feed grains were predicted by Selle et al. (2021b) where wheat’s starch digestion rate is higher than starch digestions of maize and sorghum by 1.36- and 1.56-fold factors, respectively. Therefore, broilers offered wheat-based diets may have higher amino acid catabolism in enterocytes than maize and sorghum-based diets in broiler chickens. Interestingly, low-CP, wheat-based diets resulted a 43.9 % higher proportion of uric acid-N to dietary-N ratio compared to low-CP, maize-based diets (12.3 % versus 6.9 %) in Selle et al. (2021a).

### Non-bound *versus* protein-bound amino acids

Commercial broiler diet formulations often pay less attention to the energy contribution of NBAA. Furthermore, the metabolizable energy values used for NBAA may need to be revised, as the metabolizable energy of protein-bound (PBAA) and NBAA could be different. The difference between PBAA and NBAA presumably associated with their lack of bioequivalence as discussed by Selle et al. (2022b). Moreover, Rostagno, 2017 reported the gross energy of crystalline amino acids whilst Boisen and Verstegen (2000) reported the gross energy of amino acids from intact proteins. Comparison between the estimated gross energy content of PBAA and NBAA, illustrated variations, spanning from modest to elevated values, are tabulated in Table 3**.** Moreover, energy contributions from NBAA to broiler diets were estimated in Table 4 using diet formulations in recently published wheat-based, reduced-CP studies by the Poultry Research Foundation. On average, transition of dietary-CP from 210 to 168 g/kg increased the gross energy contribution from NBAA to diet from 57.4 kcal/kg to 239.0 kcal/kg. Moreover, the contribution of NBAA to apparent metabolizable energy (AME) and nitrogen corrected apparent metabolizable energy (AMEn) in broiler chickens’ diet is expected to be different than that of PBAA. This is because NBAA generally exhibit higher digestibility coefficients (Liu et al., 2013) and have different metabolism compared to PBAAs (Ospina-Rojas et al., 2014; Ospina-Rojas et al., 2013; Selle et al., 2022b). Moreover, it was stated that dietary NBAAs and PBAAs metabolized independently at post-prandial stage of rats and human in Nolles et al. (2009).

**Table 3:** Gross energy of amino acids.

Amino acid	Gross energy (kcal/kg)		CV (%)
	Boisen and Verstegen (2000)1	Rostagno (2017)^2^	
Alanine	4,350	4,389	2.22
Arginine	5,115	4,492	9.17
Asparagine	-	2,854	-
Aspartic acid	2,892	2,854	0.94
Cysteine	4,398	4,32543	1.18
Glutamic acid	3,657	3,686	0.56
Glutamine	-	4,206	-
Glycine	3,083	3,163	1.81
Histidine	5,186	4,036	17.64
Isoleucine	6,597	6,605	0.06
Leucine	6,597	6,714	1.24
Lysine	5,617	6,204	7.02
Methionine	4,446	5,684	17.28
Phenylalanine	6,740	6,932	1.99
Proline	5,664	5,065	77.90
Serine	3,298	3,306	0.17
Threonine	4,111	4,173	1.06
Tryptophan	-	6,506	-
Tyrosine	5,951	5,990	0.46
Valine	5,975	6,026	0.60

1 Gross energy of amino acids in endogenous flow.

2 Gross energy of NBAA.

**Table 4:** Estimated gross energy contribution from dietary NBAA in wheat-based diets used in various experiments at the Poultry Research Foundation, Australia.

Item (g/kg)	Greenhalgh et al. (2022)		Chrystal et al. (2021)		Greenhalgh et al. (2020)		Macelline et al. (2023c)		Macelline et al. (2023b)				Macelline et al. (2023a)	
Diet code	1A	4D	4D	6F	5E	7G	1A	3C	1	3	2	4	1A	3C
*l*-Lysine HCl	1.51	7.24	2.36	9.72	2.94	8.60	4.20	10.7	4.24	9.19	4.96	10.1	5.64	11.0
*d,l*-Methionine	2.28	3.92	2.75	4.81	3.16	4.65	2.68	4.31	2.59	3.73	2.88	4.08	2.71	4.59
*l*-Threonine	0.63	3.22	1.59	4.93	1.40	3.93	1.68	4.53	1.78	3.92	1.99	4.21	2.67	5.22
*l*-Tryptophan	-	0.25	-	0.67	-	0.31	-	0.65	-	0.47	-	0.68	-	0.71
*l*-Valine	-	3.19	0.47	4.61	0.26	3.27	0.90	4.30	0.94	3.55	1.00	3.72	1.26	4.56
*l*-Arginine	-	4.25	-	6.99	0.19	5.39	0.69	6.77	0.80	5.43	2.14	6.95	2.29	7.78
*l*-Isoleucine	-	2.97	0.01	4.15	-	2.93	0.44	3.82	0.68	3.28	0.74	3.43	0.89	4.06
*l*-Leucine	-	3.04	-	5.39	-	4.16	-	4.84	-	3.66	0.39	4.81	-	5.32
*l*-Histidine	-	0.69	-	1.55	-	0.79	-	1.25	-	0.80	-	1.31	-	1.39
Glycine	-	-	0.41	3.95	-	4.68	0.13	5.57	0.40	2.57	0.73	2.99	0.01	6.19
*l*-Serine	-	-	0.43	4.76	-	-	-	-	0.31	2.93	0.71	3.44	-	-
*l-*Phenylalanine	-	-	-	-	-	-	-	2.53	-	-	-	1.70	0.84	6.96
*l-*Tyrosine	-	-	-	-	-	-	-	2.14	1.32	6.50	-	1.79	-	-
*l-*Proline	-	-	-	-	-	-	-	-	-	-	6.68	8.46	-	-
Dietary-CP (g/kg)	215	163	213	163	213	174	205	170	201	175	209	161	211	173
GE in diet (kcal/kg)	4,015	3,800	4,159	4,159	4,063	3,896	-	-	-	-	-	-	3,967	3,848
**GE from NBAA (kcal/kg)**	23	**153**	**38**	**258**	**41**	**196**	**55**	**268**	**67**	**237**	**122**	**308**	**84**	**304**
Portion (%)	0.60	4.02	0.92	6.21	1.00	5.03	-	-	-	-	-	-	2.11	7.89

The GE values for individual non-bound amino acids, obtained from Rostagno, 2017, were used to calculate their contribution to the GE content of each diet and to estimate the proportion of GE derived from non-bound amino acids relative to the total GE in the diets. However, Macelline et al. (2023c) and Macelline et al. (2023b) did not report the GE values of the diets; therefore, the proportion of GE contribution from non-bound amino acids could not be determined in their studies.

Rosebrough et al. (1990) revealed that dietary non-bound lysine and protein bound lysine influenced glucose and lipid metabolism differently in broiler chickens based on an *in vitro* metabolic assay. In this study, pyruvate carbon contribution for lipogenesis was significantly higher in low-CP, non-bound lysine supplemented diets than conventional-CP diets which have lysine from only intact proteins. The diets had same maize meal inclusions. Similarly, the outcomes of Aletor et al. (2000) suggested that NBAA might influence lipogenesis differently from PBAA in broiler chickens. Moreover, Nasr and Kheiri (2012) reported higher fat deposition in broiler chickens fed iso-nitrogenous diets with higher or lower lysine concentrations than in those fed a diet with standard lysine concentration. Similarly, in Yamazaki et al. (2006), excess glycine and tryptophan supplementation in the non-bound form to 190 g/kg CP diets resulted in higher fat deposition than in those fed control diets with 210 g/kg CP. However, in the same study, treatments fortified with branched chain amino acids, threonine, methionine, basic amino acids, and aromatic amino acids did not show significant increases in fat deposition compared to the control diet. Moreover, supplementation of non-bound glycine and glutamic acid resulted in higher carcass fat content in broilers offered reduced-CP diets compared to those fed control diets (Deschepper and deGroote, 1995).

The malic enzyme facilitates the conversion of malate to acetyl-co enzyme. In broiler chicken, the activity of hepatic malic enzyme is positively correlated with fatty acid synthesis, potentially leading to an increase in both body fat and abdominal fat-pad weights (Grisoni et al., 1991; Yeh and Leveille, 1969). Interestingly, Takahashi and Akiba (1995) reported that broilers fed diets with higher NBAA generated increased malic enzyme activity in the liver. These outcomes also indicate that the effects of dietary NBAAs and PBAAs on lipogenesis could differ in broiler chickens. It was reported that broilers offered reduced-CP diets with a higher-NBAA regime exhibited 11.8 % increase in fat deposition compared to those on a low-NBAA regime, despite comparable dietary starch, crude fat concentrations and feed intakes in Macelline et al. (2023b). Additionally, in Macelline et al. (2023a), dietary NBAA intakes were linearly related to abdominal fat-pad weights (r = 0.528; P = 0.029) in broiler chickens but dietary starch intakes were not related. Furthermore, dietary NBAA intakes were linearly related to plasma NH_3_ concentrations in the same study (r = 0.572; P = 0.016). Similarly, outcomes of Namroud et al. (2008) and Hofmann et al. (2019) exhibited that higher dietary NBAA increased plasma NH_3_ concentrations in broiler chickens. Likewise, higher dietary NBAA supported increased uric acid-N in excreta (Brink et al., 2022; Selle et al., 2021a). These outcomes suggest that higher NBAA intake leads to increased amino acid catabolism in the liver and higher NH₃ production, while the remaining carbon skeleton is presumably involved in *de novo* lipogenesis to some extent. A multiple linear regression was deduced from 71 data points from the studies in Table 2 where NBAA intake was positively related to abdominal fat-pad deposition (Y), but starch intake had negative impact and added fat intake did not influence the fat-pad weights at 188 g/kg of average dietary-CP concentrations (Y = 14.7 – 0.007 × Starch intake + 0.032 × NBAA intake; P < 0.001, r = 0.757). Interestingly, the quadratic relationship between dietary-CP and abdominal fat-pad weights (Y) suggests that reducing dietary CP below 188 g/kg leads to increased fat-pad deposition, as demonstrated by the equation Y = 18.2 – 0.055 × CP + 0.003 (CP – 188)² (r = 0.669; P < 0.001). This highlights a critical threshold for CP reduction, where further decreases may promote fat accumulation which might involve carbon skeletons generated from NBAA catabolism. Additionally, it is essential to recognize that elevated dietary starch ratios, along with higher intestinal starch disappearance rate ratios, also contribute significantly to the increased fat deposition observed in broilers fed reduced-CP diets (Selle et al., 2022a).

The catabolism of NBAA and PBAA in enterocytes is likely different. Interestingly, Selle et al. (2015) hypothesized that dietary NBAA may be less susceptible to catabolism in the intestinal mucosa due to their absorption in proximal segments of the small intestine, where increased glucose availability serves as an alternative energy substrate. A pig study indicated that PBAA undergo higher utilization in enterocytes than NBAA (Eugenio et al., 2023). It was demonstrated that non-bound tryptophan and lysine are less susceptible to catabolism by enterocytes compared to their protein-bound counterparts in Van der Meulen et al. (1997). Table 5 presents the influence of three dietary treatments on portal amino acid concentrations in broiler chickens, based on data from Moss et al. (2018). The results showed that increasing the concentration of dietary non-bound lysine, methionine, threonine, and valine by 3.47, 1.55, 5.62, and 1.79-fold, respectively, led to corresponding increases in their portal concentrations by 94.4 %, 68.8 %, 60.8 %, and 36.6 %. Notably, the disappearance rates of these amino acids in the small intestine were not affected by the inclusions of NBAA. Similarly, data from Golberg and Guggenheim (1962) suggested that a greater proportion of lysine from rapidly digestible protein sources enters the portal circulation than from slower-digesting proteins. Fang et al. (2010) proposed that chemical modification of NBAA could reduce their metabolism in the gut mucosa, thereby increasing their availability for systemic circulation. Therefore, in reduced-CP diets, amino acid catabolism in enterocytes appears to be minimal, resulting from a higher proportion of dietary NBAA relative to PBAA. Also, reduced-CP diets typically contain higher starch concentrations, which could be beneficial for broiler chickens by allowing more amino acids to be allocated to muscle protein synthesis rather than being utilized by enterocytes. However, this shift may negatively impact on nitrogen balance in enterocytes, potentially leading to increased utilization of arterial amino acids by enterocytes to maintain nitrogen equilibrium. Higher amino acid catabolism could potentially lead to an increase in ammonia accumulation in enterocytes. However, there is a lack of studies investigating the metabolic pathway of ammonia accumulated in enterocytes resulting from amino acid catabolism. It can be assumed that ammonia may either be released into the intestinal lumen to facilitate the nitrogen requirement of proteolytic bacteria present in the caecum, or it may be transmitted to the portal circulation for uric acid syntheses in the liver.

**Table 5:** The influence of three dietary treatments on distal ileum disappearance rates and amino acid concentrations in portal circulation (anterior mesenteric vein) as reported in Moss et al. (2018).

Amino acid	Analysed amino acid concentrations in diets, g/kg			NBAA inclusions, g/kg			Distal ileum disappearance rate, g/bird/day			Amino acid concentrations in portal circulation, mg/mL		
	1A	3C	5E	1A	3C	5E	1A	3C	5E	1A	3C	5E
Arginine	13.6	12.8	13.1	-	0.37	0.46	1.12	1.08	1.07	95.0	86.0	71.4
Histidine	5.60	5.10	5.20	-	-	-	0.44*	0.41	0.40	17.3^b^	13.1^ab^	10.1^a^
Isoleucine	9.30	8.90	8.80	-	0.93	0.97	0.73	0.70	0.66*	20.1	24.9	24.8
Leucine	16.3	14.3	14.7	-	-	-	1.27*	1.10	1.09	35.6	30.1	27.8
Lysine	12.4	12.5	12.9	0.76	2.64	2.73	0.99	1.04	1.05	39.5^a^	76.8^b^	62.3^b^
Methionine	5.20	5.60	5.70	2.66	4.12	4.12	0.45*	0.52	0.51	12.8^a^	21.6^b^	17.4^ab^
Phenylalanine	10.1	9.10	9.40	-	-	-	0.81	084	0.85	25.5	25.9	24.1
Threonine	8.60	8.30	8.60	0.29	1.63	1.66	0.61	0.59	0.60	51.0^a^	82.0^b^	66.1^ab^
Valine	10.4	10.2	9.70	-	1.79	1.82	0.80	0.79	0.71*	30.6^a^	41.8^b^	42.6^b^
Alanine	9.10	10.1	11.0	-	-	2.47	0.66	0.57*	0.66	108.9	112.9	122.9
Aspartic acid	21.0	19.5	21.3	-	-	3.11	1.33	1.10*	1.34	45.3	47.3	46.5
Cysteine	2.50	2.60	2.50	-	-	-	0.13	0.14^⁎⁎^	0.12*	67.3	80.4	74.1
Glutamic acid	35.6	32.2	35.9	-	-	7.30	2.84	2.55*	2.80	214	243	229
Glycine	8.40	8.10	8.40	-	-	1.46	0.60	0.58	0.58	59.8	52.8	60.4
Proline	10.9	10.8	11.0	-	-	3.16	0.81	0.84	0.82	58.0	49.1	61.0
Serine	10.1	9.80	10.1	-	-	1.94	0.74	0.75	0.75	70.9	83.1	78.3

1A, 3C and 5E are three of six dietary treatments in Moss et al. (2018).

The asterisks indicate the significantly difference means for amino acid disappearance rates in distal ileum based on six dietary treatments in Moss et al. (2018).

abc Means within rows not sharing a common suffix are significantly different at the 5 % level of probability.

## Potential challenges and future directions

This review suggests that minimizing the utilization of dietary amino acids as an energy substrate in enterocytes and the liver may enhance protein utilization, thereby improving growth performance and overall productivity in broiler chickens. The efficiency of amino acid conversion to muscle protein is particularly critical due to limited amino acid pool in reduced-CP diets than conventional-CP diets. Moreover, amino acid utilization as an energy source more likely differs between conventional- and reduced-CP diets, primarily due to variations in starch and protein digestion dynamics. Glutamate serves as the primary energy source for enterocytes in broiler chickens; however, its lower concentrations in reduced-CP diets may lead to increased catabolism of essential amino acids to meet energy demands in enterocytes. This challenge could be more pronounced in wheat-based, reduced-CP diets compared to maize- or sorghum-based counterparts. The rapid digestibility of wheat starch may exacerbate amino acid catabolism in enterocytes, whereas the slower digestibility of maize and sorghum starches may mitigate this effect up to some extent. Additionally, reduced-CP diets tend to result in greater amino acid catabolism in the liver than conventional-CP diets, leading to elevated uric acid synthesis and potentially increased *de novo* lipogenesis from the α-keto acids of deaminated amino acids. Both uric acid synthesis and *de novo* lipogenesis are energy-intensive processes. While α-keto acids can be fully oxidized in the liver to generate ATP, this process is regard as inefficient due to increased heat increments compared to the oxidation of glucose and fatty acids.

The possibility of dietary manipulation to reduce amino acid utilization as an energy substrate has been hinted by previous studies. However, there is lack of suggestions on dietary strategies have been reported in the literature in terms of diminishing the metabolic cost of amino acid utilization for energy. Limiting the amino acid catabolism in the liver and enterocytes remains a challenge. Amino acid requirements in broilers offered reduced-CP diets appear to differ substantially from those in birds fed conventional-CP diets due to the varying degrees of amino acid catabolism in enterocytes and liver. Similarly, the type of grain used in the diet influences amino acid utilization, as starch digestion dynamics impact amino acid catabolism. This underscores the need for amino acid recommendations that account for both dietary CP concentrations and the primary feed grain used. In the context of reduced-CP diets, supplementation with Glu, Gln, aspartate, and proline may be beneficial, as these amino acids serve as key energy substrates for enterocytes and has low concentrations in reduced-CP diets. The choice of feed grain exerts a greater influence in reduced-CP diets than in conventional-CP diets due to their higher inclusion rates. Given that rapid starch digestion increases amino acid catabolism in enterocytes, dietary strategies such as whole grain feeding may enhance post-enteral amino acid availability by moderating starch digestion rates, particularly in wheat-based, reduced-CP diets. Manipulating amino acid catabolism and *de novo* lipogenesis in the liver through dietary interventions is complex. However, optimizing the balance of NBAAs PBAAs in broiler diets may help minimize post-enteral amino acid catabolism by reducing protein imbalances at the site of protein synthesis. This is especially relevant for reduced-CP diets, which typically contain higher levels of NBAAs than conventional-CP diets. Further research is warranted to establish the optimal NBAA-to-PBAA ratio, considering the specific feed grain composition of the broiler diets.

## Conclusion

Amino acid catabolism for energy production in broiler chickens represents a metabolic cost, as the primary role of amino acids is to support protein synthesis. While the precise mechanisms driving amino acid catabolism have not been fully elucidated, current evidence suggests that it may be influenced by the rate of starch digestion and the dietary balance between PBAA and NBAA. This issue is particularly relevant in broilers fed reduced-CP diets, where the available amino acid pool is more limited compared to conventional-CP diets. Enterocytes and the liver remain key sites of amino acid catabolism, and further investigation is needed to better understand the underlying factors and how they can be managed through dietary strategies.

## Disclosures

The authors declare that they have no financial and personal relationships with other people or organizations that can inappropriately influence their work and that there is no professional or other personal interest of any nature or kind in any product, service, and/or company that could be construed as influencing the content of this article.

## Declaration of competing interest

The authors declare that they have no financial and personal relationships with other people or organizations that can inappropriately influence their work and that there is no professional or other personal interest of any nature or kind in any product, service, and/or company that could be construed as influencing the content of this article.
